# Mitochondrial Function in Peripheral Blood Mononuclear Cells (PBMC) Is Enhanced, Together with Increased Reactive Oxygen Species, in Severe Asthmatic Patients in Exacerbation

**DOI:** 10.3390/jcm8101613

**Published:** 2019-10-03

**Authors:** Carole Ederlé, Anne-Laure Charles, Naji Khayath, Anh Poirot, Alain Meyer, Raphaël Clere-Jehl, Emmanuel Andres, Frédéric De Blay, Bernard Geny

**Affiliations:** 1 Pôle de Pathologie Thoracique, Service de Pneumologie, Nouvel Hôpital Civil, 1, Place de l’Hôpital, FHU OMICARE Université de Strasbourg, 67000 Strasbourg, France; carole.ederle01@gmail.com (C.E.); naji.khayath@gmail.com (N.K.); anh.poirot@chru-strasbourg.fr (A.P.);; 2 Fédération de Médecine Translationnelle de Strasbourg (FMTS), Faculté de Médecine, Equipe d’Accueil 3072, «Mitochondrie, Stress Oxydant, et Protection Musculaire», 11 Rue Humann, Université de Strasbourg, 67000 Strasbourg, France; anne.laure.charles@unistra.fr (A.-L.C.); alain.meyer1@chru-strasbourg.fr (A.M.); Raphael.clere-jehl@chru-strasbourg.fr (R.C.-J.); 3 Service de Physiologie et d’Explorations Fonctionnelles, Hôpitaux Universitaires de Strasbourg, Nouvel Hôpital Civil, 1 Place de l’Hôpital, 67091 Strasbourg CEDEX, France; 4 Service de Médecine Interne, Diabète et Maladies Métaboliques, Pôle M.I.R.N.E.D., Hôpitaux Universitaires, 67000 CHRU Strasbourg CEDEX, France; Emmanuel.andres@chru-strasbourg.fr

**Keywords:** asthma, exacerbation, reactive oxygen species, PBMC, mitochondrial function

## Abstract

Asthma is a chronic inflammatory lung syndrome with an increasing prevalence and a rare but significant risk of death. Its pathophysiology is complex, and therefore we investigated at the systemic level a potential implication of oxidative stress and of peripheral blood mononuclear cells’ (PBMC) mitochondrial function. Twenty severe asthmatic patients with severe exacerbation (GINA 4–5) and 20 healthy volunteers participated at the study. Mitochondrial respiratory chain complexes activities using different substrates and reactive oxygen species (ROS) production were determined in both groups by high-resolution respirometry and electronic paramagnetic resonance, respectively. Healthy PBMC were also incubated with a pool of plasma of severe asthmatics or healthy controls. Mitochondrial respiratory chain complexes activity (+52.45%, *p* = 0.015 for V_ADP_) and ROS production (+34.3%, *p* = 0.02) were increased in asthmatic patients. Increased ROS did not originate mainly from mitochondria. Plasma of severe asthmatics significantly increased healthy PBMC mitochondrial dioxygen consumption (+56.8%, *p* = 0.031). In conclusion, such asthma endotype, characterized by increased PMBCs mitochondrial oxidative capacity and ROS production likely related to a plasma constituent, may reflect activation of the immune system. Further studies are needed to determine whether increased PBMC mitochondrial respiration might have protective effects, opening thus new therapeutic approaches.

## 1. Introduction

Asthma is a common disease that affects 300 million people worldwide (1 in 10 children and 1 in 12 adults) resulting in a substantial morbidity and an important annual healthcare expenditure. The disease is characterized by chronic inflammation of the conducting airway resulting in bronchial obstruction, mucus overproduction, airway remodeling and bronchial hyper-responsiveness [1,2].

Different mechanistic pathways are involved in such complex pathology. Locally, generation of oxidative stress with increased reactive oxygen species (ROS) production and mitochondrial dysfunction have been observed (bronchus, airway epithelium) in murine models of ovalbumin-induced asthma and in bronchial epithelial cell cultures [3,4,5,6,7,8]. Increased ROS production results in oxidative lipid peroxidation, protein and DNA damages (single-stranded and double-stranded breaks) [9], thought to aggravate the airway inflammation and to play a role in bronchial smooth muscle impairment and mucus secretion. Damaged antioxidant defense mechanisms, altered homeostasis of the airway surface liquid and acute or chronic bacterial airway infections also seem to aggravate oxidative stress in asthma [10]. Several studies highlighted an imbalance between oxidative species and antioxidant mechanisms in asthma. A decrease of superoxide dismutase, gluthation peroxidase or catalase activity was correlated with an aggravation of bronchial obstruction and asthma severity [11,12,13,14,15]. A self-entertaining phenomenon of inflammation and oxidative stress production occurs and damage-associated molecular patterns (so called alarmins), activate immune cells and their specific tissue [16].

In turn, immune cells of asthmatic patients exposed to an allergen, generate ROS, such as hydroxyl radicals, superoxide, peroxydes, peroxynitrite and nitric oxide [9]. This ROS generation was observed in sputum, exhaled breath condensates and bronchoalveolar lavages (BAL) of asthmatic patients, especially in severe asthma [11,12,13,14].

Besides this local characterization of the asthma mechanism, there are relatively few data focused on a potential involvement of mitochondrial function of inflammatory circulatory cells. However, dysfunction of the mitochondrial respiratory chain is likely to play a role in the initiation and progression of other inflammatory pathology such as cardiovascular diseases and anaphylactic choc [17,18,19,20]. Furthermore, impairments in mitochondrial function and/or elevation of ROS production have been found in allergic diseases such as atopy, atopic dermatitis and allergic rhinitis [3,4,21,22]. Recently, studying systemic involvement in local allergic rhinitis, we observed an impaired mitochondrial function in peripheral blood mononuclear cells (PBMC) 6 hours after an allergen challenge in patients with allergic rhinitis [23].

In the present study, therefore, we tested the hypothesis that peripheral blood mononuclear cell might demonstrate impaired mitochondrial function together with increased ROS production in severe asthmatic patients with severe exacerbation, as compared to healthy subjects.

## 2. Population and Methods

### 2.1. Patients and Study Design

Twenty healthy volunteers and 20 severe asthmatic patients experiencing severe exacerbation were enrolled in a prospective and controlled study. Severe asthma was defined as a stage 4 or 5 of GINA classification and severe exacerbation as a need for hospitalization or for systemic steroids (or its increase) for more than 3 days [2]. Exclusion criteria were active smoking, smoking cessation <1 year; ancient smoking >10 packs a year, current depression, cardiovascular insufficiency or severe sepsis.

The study design included the clinical characterization of patients and, peripheral blood mononuclear cells mitochondrial respiratory chain complexes’ activities and ROS production in both control subjects and asthmatic patients. Further, after determining an eventual contribution of mitochondria to ROS production, we analyzed the effects of plasma on healthy PBMC. Thus, PBMC from healthy volunteers were placed in contact with heterologous platelet poor plasma of healthy volunteers or severe asthmatics experiencing severe exacerbation.

The study was approved by the ethics committee of the Strasbourg University Hospital (number 2016-69) and informed consent was obtained from each patient.

### 2.2. Peripheral Blood Mononuclear Cells (PBMC) Isolation 

Thirty mL of venous blood was sampled in Sodium Heparinate tubes for each patient. 1mL was kept (on ice) for the study of ROS production. The remaining part of the sample was used to separate peripheral blood mononuclear cells (PBMC) and plasma. Briefly, blood was placed on a ficoll density gradient (Eurobio, Lymphocytes separation medium, Courtabeauf France, France) and centrifuged (2100 rpm, 25 min, 18 °C, without brakes). PBMC (lymphocytes and monocytes) were sampled, washed in a DPBS solution (Dulbecco’s Phosphate Buffer Saline 0067M, Hyclone, South Logan, UT, USA) and centrifuged (1600 rpm, 10 min, 18 °C, without brakes). Finally, PBMC were counted by flow cytometry (Muse Cell Analyser, Merck Millipore, Darmstadt, Germany).

### 2.3. Mitochondrial Respiration 

The study of the mitochondrial respiratory chain was performed with a high resolution oxygraph (Oxygraph-2k; Oroboros Instruments, Innsbruck, Austria) at 37 °C. 2.5 × 10^6^ PBMC /mL were introduced in the Oxygraph-2k’s chamber with continuous stirring. The dioxygen consumption was analyzed using the DatLab software 4.3 (Oxygraph-2k; Oroboros Instruments, Innsbruck, Austria). At the beginning, cell membranes were permeabilized with saponine (125 µg/mL), and complex I was activated with glutamate (5 mM), and malate (2 mM), this step is the basal dioxygen consumption.

After reaching a steady state for V0, different substrates and inhibitors were introduced in the oxygraph’s chamber. ADP (2 mM) induced the activation of ATP synthase (mitochondrial Complex V) and allowed the study of mitochondrial complexes I, III, IV, V. Then, succinate (25 mM) was introduced, an activator of the mitochondrial Complex II, for the study of mitochondrial Complexes I, II, III, IV, V. The addition of rotenone (0.5 μM) allowed analysis of mitochondrial Complexes II, III, IV, V by inhibiting the Complex I. TMPD/ascorbate (0.5 mM/0.5 mM) was then added to give electrons to the Complex IV, so allowed preferentially the complex IV. The result was expressed in pmol/s/10^6^cell.

### 2.4. Reactive Oxygen Species (ROS) Production

#### 2.4.1. Measurement of Superoxide Anions in Blood

ROS production in venous blood was assessed by exploring the superoxide anions production, using electron paramagnetic resonance (EPR), (E-scan, Bruker-Biospin, Rheinstetten, Germany) at 37 °C, as previously described [24]. Briefly, 1 mL venous blood was kept on ice in order to perform the analysis 1 hour after sampling; 25 µL of blood were mixed with spin probe CMH (1-hydroxy-3-methoxycarbonyl-2,2,5,5-tetramethylpyrrolidine HCl, 200 µM). Then, the mixture was introduced in a glass EPR capillary tube (Noxygen Science Transfer & Diagnostics, Elzach, Germany), and then placed inside the cavity of the e-scan spectrometer (Bruker, Rheinstetten, Germany) for data acquisition. Detection of ROS production was conducted using BenchTop EPR spectrometer E-SCAN under the following EPR settings: center field *g* = 3477,452; sweep width 60 G; microwave power 21.85 mW; modulation amplitude 2.4 G; time constant 40.96 ms; conversion time 10.24 ms; number of lag curve points 6. The EPR signal is proportional to the unpaired electron numbers. The result was expressed in µmol/min.

#### 2.4.2. Mitochondrial ROS measurement

We determined mitochondrial PBMC ROS production using a fluorescent probe MitoSOX, a red mitochondrial superoxide indicator for live cell imaging (Molecular Probes; Life technologies) [25]. MitoSOX (Invitrogen, Eugene, Oregon, USA) (5 µM) was incubated with 2.5 × 10^6^ PBMC diluted in DPBS, for 10 min at 37 °C. Then, cells were washed and centrifuged (1600 rpm, 10 min). Excitation was set at 510 nm and emission at 580 nm A positive control group of mitochondrial ROS production was elaborated with Antimycin. All the experiments were achieved in a light-protected environment. The result was expressed in AU of fluorescence.

### 2.5. Mitochondrial Dioxygen Consumption of Healthy PBMC in Contact with Heterologous Plasma

PBMC from 7 healthy volunteers were extracted following the same technique as presented above. Then 2 × 10^6^ cells/mL were placed into each oxygraph’s chamber with a pooled heterologous platelet poor plasma. In one chamber, plasma came from a pool of 6 healthy volunteers, and in the other one, plasma came from a pool of 6 severe asthmatics experiencing severe exacerbation.

Before introducing the PBMC, the basal respiration of both pooled plasmas was recorded in the oxygraph’s chambers (Oxygraph-2k; Oroboros Instruments, Innsbruck, Austria) at 37 °C.

After 6 h exposure to heterologous plasma, PBMC basal consumption of dioxygen (V0) was measured and different substrates and inhibitors were added in order to study the mitochondrial respiratory chain complexes’ activities. Using plasma as the respiratory solution, the uncoupling protocol was more adapted than the classic one. Therefore, an ATP-synthase inhibitor (oligomycine 1.25 µmol/L) was added, to induce a dioxygen consumption mainly due to the leakage of protons through the inner mitochondrial membrane [26]. Then, the uncoupler carbonyl cyanide 4-(trifluoro-methoxy)phenylhydrazone (FCCP 40 µM) was added in order to measure maximal oxygen flux. Rotenone (2 µM) was added to inhibit the complex I. Finally, Complex III was inhibited by antimycine (1.85 µM). Dioxygen consumption was calculated by subtracting the basal plasma respiration as previously described [20,26,27]. The result was expressed in pmol/s/10^6^cell.

Simultaneously, ROS production was measured by the evolution of using fluorescent probe Amplex Red (20 µM) and Horseradish peroxidase (HRP, 1 U/mL). We also calculated the free radical leak (FRL) corresponding to the fraction of electrons which reduces O_2_ to ROS in the mitochondrial respiratory chain (percentage of free radical leak) instead of reaching cytochrome oxidase to reduce O_2_ to water [28]. Because two electrons are needed to reduce one mole of O_2_ to H_2_O_2_, whereas four electrons are transferred in the reduction of one mole of O_2_ to water, the FRL (percent) was calculated as the rate of H_2_O_2_ production divided by twice the rate of O_2_ consumption, and the result was multiplied by 100 [29].

### 2.6. Statistical Analysis

All values are presented as mean ± standard error of the mean (SEM). Qualitative variables were described as numbers and percentages. When values followed the normality curve, Student’s test was used. But, if values did not follow the normality curve, a non-parametric Mann–Whitney test was used to compare the control and the asthmatics groups. Concerning data obtained with PBMC exposed to pooled heterogenous control plasma or pooled heterogenous asthmatic plasma, a non-parametric paired test was realized (Wilcoxon test). All analysis were performed with GraphPad Prism 5 (Graph Pad Software, Inc., San Diego, CA, USA). Statistical significance was determined as *p* < 0.05.

## 3. Results

### 3.1. Clinical Characteristics of the Subjects

Twenty patients (mean age 50.95 ± 4.06 years, 5 men and 15 women) were admitted for exacerbation of severe asthma at the Strasbourg University Hospital with a need for hospitalization or systemic steroids for more than 3 days. The control group included 20 healthy volunteers (mean age 50.10 ± 3.77 years, 6 men and 14 women) with no medical history of pulmonary or atopic diseases.

Subjects’ characteristics are summarized in Table 1. There was no difference in age, sex and smoking between severe asthmatic patients and the control group. Asthmatic patients had a body mass index (BMI) of 28.01 ± 1.47 kg/m^2^ versus 23.0 ± 0.72 kg/m^2^ in the control group (*p* = 0.0040). Severe asthmatic patients had mainly an uncontrolled allergic asthma. Fourteen patients presented with atopic and eosinophilic asthma whereas 2 patients had a non-atopic and eosinophilic asthma and 4 patients had non-Th_2_ mediated asthma. 14 patients had an associated allergic rhinitis, 8 had a basal FEV1 < 80% of the maximal theoretical value. The main cause of exacerbation was bronchial and pulmonary infection and 9 patients were treated by steroids for more than 24 h at the time of inclusion and blood sampling.

### 3.2. Mitochondrial Respiratory Chain Complexes’ Activities Are Enhanced in Asthmatic Patients

**Figure 1:**
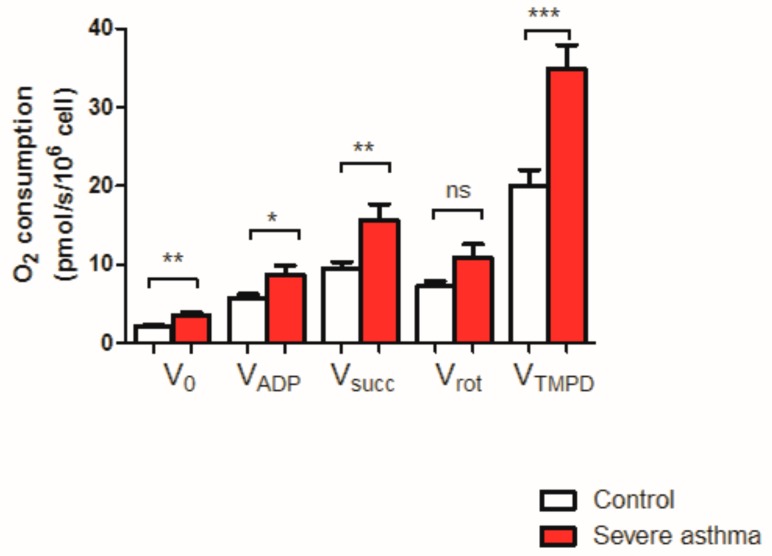
Peripheral blood mononuclear cells (PBMC) mitochondrial respiration in control and severe asthmatic patients. * *p* < 0.05, ** *p* > 0.01, *** *p* < 0.001. ns: non significant.

The maximal mitochondrial respiratory chain activity was significantly increased in patients with severe asthma experiencing severe exacerbation in comparison with the control group (Figure 1). Data using specific substrates are presented below.

#### 3.2.1. Basal Consumption of Dioxygen (V_0_)

V0 was increased in severe asthmatic patients in comparison with the control group (3.51 ± 0.43 and 2.10 ± 0.22 pmol/s/10^6^cell respectively, +67.1%, *p* = 0.005).

#### 3.2.2. Mitochondrial Complexes I + III + IV + Vactivities

V_ADP_ was significantly increased in the severe asthma group compared to the control group (8.78 ± 1.11 vs. 5.76 ± 0.48 pmol/s/10^6^cell respectively in asthmatic patients and in control subjects, +52.4%, *p* = 0.015).

#### 3.2.3. Mitochondrial Complexes I + II + III + IV + Vactivities

Vsucc was also significantly increased in the severe asthma group compared to the control group (15.64 ± 2.06 and 9.48 ± 0.91 pmol/s/10^6^cell, +64.97%, *p* = 0.007).

#### 3.2.4. Inhibition of the Mitochondrial Complex I by Rotenone

Rotenone injection showed no significant difference between both groups but the combined activity of the Complexes II + III + IV + V tended to be increase in the severe asthmatic patients (10.89 ± 1.70 and 7.22 ± 0.67 pmol/s/10^6^cell respectively in asthmatic patients and in control subjects, +50.83%).

#### 3.2.5. Complex IV Activity

V_TMPD/ascorbate_ showed an increase of cytochrome C oxidase’s activity in asthmatic patients (34.87 ± 2.99 and 20.03 ± 2.02 pmol/s/10^6^cell, +74.09%, *p* = 0.0003).

V_0_ corresponds to the basal O_2_ consumption, with glutamate, and malate as substrates. V_ADP_ corresponds to the ADP-stimulated respiration, with glutamate and malate as substrates. V_succ_ represents the activation of all complexes (I, II, III, IV, V). V_ROT_ represents the complexes II, III, IV, V activities. V_TMPD_ corresponds to the complex IV contribution. Results are expressed as means ± SEM. *n* = 20 per group.

**Figure 2:**
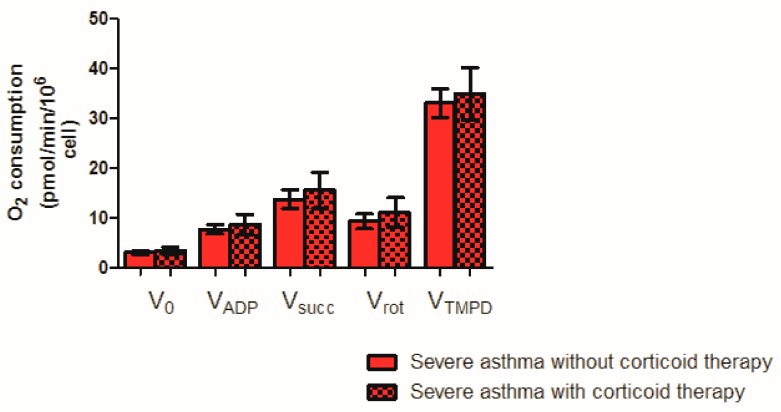
PBMC mitochondrial respiration in severe asthmatic patients treated or not with corticoids.

**Figure 3:**
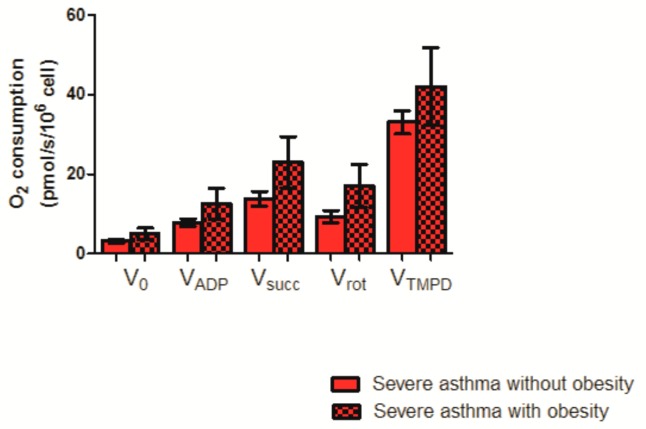
PBMC mitochondrial respiration in severe asthmatic patients, without or with a BMI ≥ 30 kg/m^2^.

To further investigate a possible effect of corticosteroid or obesity, we analyzed PBMC mitochondrial respiration in asthmatic patients with and without corticosteroid (Figure 2) and with and without a BMI ≥ 30 kg/m^2^ (Figure 3).

Concerning the corticosteroids, no significant difference was observed whatever the mitochondrial respiratory chain complex studied between patients treated or not with systemic steroids for more than 24 h.

V_0_ corresponds to the basal O_2_ consumption, with glutamate, and malate as substrates. V_ADP_ corresponds to the ADP-stimulated respiration, with glutamate and malate as substrates. V_succ_ represents the activation of all complexes (I, II, III, IV, V). V_ROT_ represents the complexes II, III, IV, V activities. V_TMPD_ corresponds to the complex IV contribution. Results are expressed as means ± SEM. *n* = 11 without and 9 with corticoid, respectively.

Moreover, although mitochondrial respiration tended to increase in severe asthmatics patients with a BMI ≥ 30 kg/m^2^ as compared to patients with a BMI < 30 kg/m^2^, this increase failed to reach statistical significance (23.01 ± 6.43 vs. 13.80 ± 1.88 pmol/s/10^6^cell, *p* = 0.09 respectively for V_succ_, and 17.07 ± 5.43 vs. 9.35 ± 1.51 pmol/s/10^6^cell, *p* = 0.08, for V_rot_).

V_0_ corresponds to the basal O_2_ consumption, with glutamate, and malate as substrates. V_ADP_ corresponds to the ADP-stimulated respiration, with glutamate and malate as substrates. V_succ_ represents the activation of all complexes (I, II, III, IV, V). V_ROT_ represents the complexes II, III, IV, V activities. V_TMPD_ corresponds to the complex IV contribution. Results are expressed as means ± SEM. *n* = 16 without and 4 with obesity, respectively.

### 3.3. Reactive Oxygen Species Are Increased in the Blood of Asthmatic Patients

**Figure 4:**
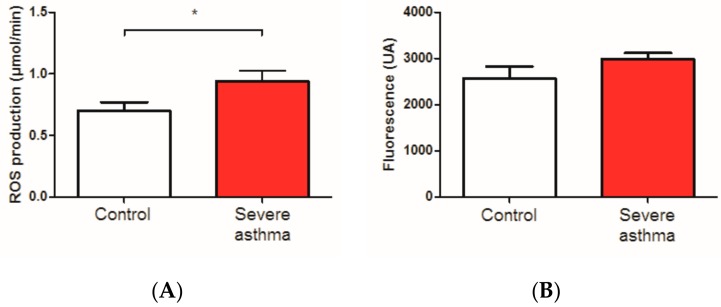
Reactive oxygen species (ROS) in control and severe asthmatic patients experiencing severe exacerbation. (**A**): total ROS level on whole blood was obtained using electron paramagnetic resonance. *n* = 16 per group. (**B**): ROS level on PBMC was detected by MitoSOX-based spectrofluorimetry detection. Results are expressed as means ± standard error of the mean (SEM). *n* = 8 per group. * *p* < 0.05.

ROS were significantly increased in the venous blood of severe asthmatic patients in comparison with the control group (0.94 ± 0.08 vs. 0.70 ± 0.07 µmol/min respectively +34.3%, *p* = 0.02, *n* = 16 per group, Figure 4A). To investigate the origin of such ROS, we determined the mitochondrial ROS production.

Mitochondrial ROS production tended to be increased in severe asthmatic patients experiencing severe exacerbation and the control group (2983.0 ± 135.8 and 2571.0 ± 252.2 UA respectively, *p* = 0.07, *n* = 8 per group, Figure 4B).

Systemic steroid treatment > 24 h and obesity did not influence the ROS production.

### 3.4. Effect of Heterologous Plasma of Healthy or Asthmatic Subjects on Mitochondrial Dioxygen Consumption and of Healthy PBMC

**Figure 5:**
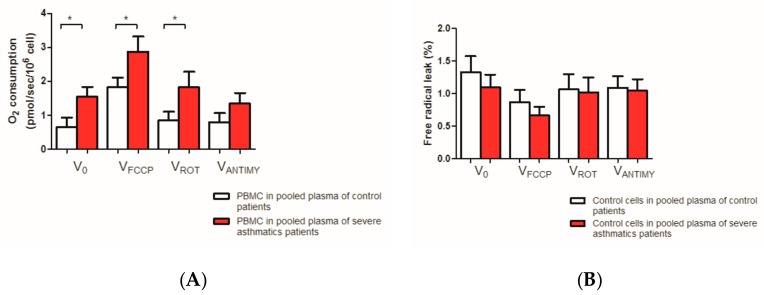
Effect of healthy and asthmatic plasma on PBMC mitochondrial respiration and free radical leak. (**A**) Mitochondrial respiration after different substrates injected. (B) Free radical leak. PBMC: peripheral blood mononuclear cell. FCCP: carbonyl cyanide 4-(trifluoro-methoxy)phenylhydrazone, ROT: rotenone, ANTIMY: antimycin. Results are expressed as means ± SEM. *n* = 7 per group. * *p* < 0.05.

Investigating whether plasma of asthmatic patients *per se* might modulate PBMC mitochondrial function, we observed using different substrates, an enhanced mitochondrial respiration of PBMC in presence of asthmatic plasma as compared to normal plasma (*n* = 7 per group, Figure 5A).

Thus, basal dioxygen consumption Vo was significantly increased in severe asthmatic plasma (1.55 ± 0.29 and 0.64 ± 0.28 pmol/s/10^6^ cell, +142.2%, *p* = 0.016).

After ATP synthase inhibition mitochondrial respiration was activated by an uncoupler (FCCP). The Maximal mitochondrial respiratory chain activity was significantly increased in healthy PBMC in contact with pooled plasma from severe asthmatic patients in comparison with healthy PBMC in contact with pooled control plasma (2.87 ± 0.44 and 1.83 ± 0.28 pmol/s/10^6^cell, +56.8%, *p* = 0.031).

Similarly, when the complex I was inhibited by rotenone injection mitochondrial activity was significantly increased in healthy PBMC in contact with pooled plasma from severe asthmatic patients in comparison with healthy PBMC (1.82 ± 0.45 and 0.84 ± 0.26 pmol/s/10^6^cell, +116.7%, *p* = 0.016).

Finally, when the complex IV was inhibited by antimycin injection, mitochondrial activity tended to increase in healthy PBMC in contact with pooled plasma from severe asthmatic patients as compared to the control group (1.34 ± 0.45 and 0.80 ± 0.26 pmol/s/10^6^cell, *p* = 0.297, +67.5%)

The free radical leak was not statistically increased in healthy PBMC in contact with pooled plasma from severe asthmatic patients, in comparison with the control one. These results are presented in Figure 5B.

## 4. Discussion

The main result of this study is to demonstrate that mitochondrial respiratory chain complexes’ activities of PBMC are stimulated in severe asthmatic patients with severe exacerbation, and that such enhancement is associated with increased reactive oxygen species. Furthermore, the plasma of asthmatic patients—but not of healthy subjects—appears responsible for PBMC mitochondrial oxidative capacity enhancement.

Asthma is a frequent and severe disease affecting a large proportion of the human population, including children and young people and implying a tremendous socioeconomic burden. The pathophysiology of asthma and the balance between oxidant/antioxidant is the subject of intense ongoing research and some studies have explored the local role of mitochondria in atopic diseases [1,2,3,4,5,6,7,8,9,16,29]. However, still little is known on the connection between mitochondria and the immune response in asthma, especially in peripheral blood.

Interestingly, our study shows that severe asthmatic patients with severe exacerbation had a significant increase in their PBMC mitochondrial respiratory chain activity, in comparison with the control group. These data contrast with a previous report showing that acute nasal allergen challenge induces mitochondrial dysfunction of peripheral blood mononuclear cells in allergic rhinitis [23] and with the fact that PBMC mitochondrial respiration is generally impaired in cardiovascular diseases [18,19]. Thus, the increase observed in asthmatic patients is particularly interesting. In fact, peripheral blood immune cells like T and B lymphocytes or monocytes (PBMC) were activated in severe asthmatic patients with severe exacerbation [1]. At the mitochondrial level, this activation was expressed by an increased maximal function of the mitochondrial respiratory chain. Interestingly, this is consistent with data obtained on PBMC mitochondrial function in septic shock, often showing an increased mitochondrial respiration after an early impairment [20,30]. Such a response was thought to compensate for the initial mitochondrial dysfunction [30].

The underlying mechanisms still deserve further study but oxidative stress might play a key role. In our study, severe asthmatic patients with severe exacerbation had a significant elevation of ROS in their blood. These ROS did not seem to be mainly produced by mitochondria since the Mitosox analysis was similar in both groups and since the FRL showed no statistically significant difference in the number of O_2_ molecules used for the production of ROS in the mitochondria between the two groups. Thus, as previously reported in asthma, reactive oxygen species could have been produced by NADPH-oxidase and/or by xanthine oxidase rather than by the mitochondrion [31,32].

Obesity might also have played a role since there is a link between mitochondrial dysfunction, obesity and asthma or metabolic syndrome [33,34]. Accordingly, PBMC mitochondrial respiration tended to be increased in asthmatic patients presenting with a BMI ≥ 30kg/m^2^. Although not statistically significant and focused only on several respiratory chain complexes, this result supports further studies on a potential relationship between obesity *per se* and the mitochondrial respiration of circulating cells.

Similarly, corticosteroids might modulate PBMC mitochondrial respiration. Indeed, steroids mediated apoptotic signals in human eosinophils through a mitochondrial pathway [35]. However, in our study, no significant change was observed when comparing asthmatic patients with and without the corticoid therapy.

Finally, we tested whether patients with eosinophilia and hyper-eosinophilia, defined by a blood count of ≥300 and ≥500/mm^3^ respectively, presented with exacerbated PBMC mitochondrial respiration as compared to asthmatic patients without eosinophilia. No difference was observed between both groups, suggesting the eosinophilia might not be a key factor associated with an enhanced PBMC mitochondrial respiration.

The clinical significance of such enhanced mitochondrial respiratory chain complexes’ activities in severe asthmatic patients in exacerbation remains to be further investigated. However, recent studies suggest that during the activation of the adaptive immune response, the shift of dendritic cell’s mitochondrial respiration toward either a glycolytic or an oxidative metabolism might be related to a pro-inflammatory or to an anti-inflammatory differentiation of T cells, respectively. Thus, a reduced mitochondrial respiration seemed to be associated with pro-inflammatory effects whereas an increased dendritic cell’s mitochondrial respiration might antagonize TLR effects [36,37,38]. Therefore, one might hypothesize that the enhanced mitochondrial respiration we observed could reflect a trend toward a potentially beneficial anti-inflammatory effect.

Accordingly, besides their well-known deleterious effects corresponding to oxidative stress, ROS signaling might also allow protective effects through mitochondrial biogenesis and anti-oxidant system enhancements [39]. In asthma, ROS could have a different role depending on their producing cells. In epithelial and in bronchial smooth muscle cells, ROS likely induce local tissue damages and aggravate inflammation [9,10]. In peripheral blood, the increased ROS was observed without concomitant mitochondrial dysfunction, and could participate in the activation of circulating T lymphocytes, potentially playing a role in the “homing” of immune cells to the inflammatory upper airways [16].

Interestingly, recent data support a link between antigen-specific IgE receptor expression on PBMC and atopic asthma within school-aged children [40]. Furthermore, basophil counts in PBMC populations during childhood acute wheeze/asthma were associated with future exacerbations [41]. Whether the mitochondrial function of PBMC could be used in the future as a biomarker in severe asthma or for a targeted therapy cannot be inferred from our results. But, these data emphasize the potential interest of analyzing PBMC metabolic characteristics, in conjunction with the search of other potential biomarkers in severe asthma [42,43,44].

### Limitations of the Study

Although 20 subjects in each group allowed us to discover interesting characteristics of asthmatic patients, studies with a greater number of subjects will be useful to further investigate the underlying mechanisms. In this context, a study specifically directed toward obese patients and a longer follow-up in order to characterize the patients after the exacerbation will be very interesting for better defining the clinical significance of these data.

## 5. Conclusions

This study further supports the concept of crosstalk between the mitochondrion and the immune system in asthma. The enhanced mitochondrial respiration observed in the PBMC of patients with severe asthma in exacerbation, likely related to a plasmatic factor potentially including ROS, might participate in the patient’s defense mechanisms. However, this issue still has to be investigated and further studies are needed to determine whether circulating cells’ mitochondrial function might be used as a biomarker in asthma and whether maintaining healthy mitochondria in asthmatic patients’ PBMC might be an interesting therapeutic avenue, as proposed in lung injury [16,45]. Thus, further longitudinal studies exploring the systemic aspects of mitochondrial function and the ROS production in asthma appear warranted.

## Figures and Tables

**Table 1:** Demographic, clinical, and biological characteristics of the patients.

	Control Group (*n* = 20)	Severe Asthmatic Patients (*n* = 20)
Mean age (years)	50.10 ± 3.77	50.95 ± 4.06
Women	14	15
Male	6	5
Mean body mass index (BMI) (kg/m^2^)	23.0 ± 0.72	28.01 ± 1.47
Mean smoking habits (PA)	1.70 ± 0.80	1.90 ± 0.69
Atopy	0	16
Medical history:		
Diabetes	1	4
Arterial hypertension	3	8
Venous thrombosis/pulmonary embolism	0	1
Other lung diseases	0	2
Neoplasia	0	1
Acute coronary syndrom with preserved LVEF	2	1
Blood eosinophil (Normal values: <600/mm^3^)		
<300/mm^3^		9
300–500/mm^3^		2
500–1000/mm^3^		9
Blood neutrophils (Normal values: 1500–7500/mm^3)^		
<7500/mm^3^		16
>7500/mm^3^		4

BMI: Body mass index, PA: packs a year, LVEF: left ventricular ejection fraction.

## Abbreviations

ADP	adenosine Di-phosphate
ATP	adenosine Tri-phosphate
AU	Arbitrary unit
FEV1	Forced Expiratory Volume
GINA	global initiative for asthma
NADPH	Nicotinamide adénine dinucléotide phosphate
TMPD	’N, N, N’N’-tétraméthyl-1,4-phénylènediamine
